# Diffusion tensor imaging detects chronic microstructural changes in white and gray matter after traumatic brain injury in rat

**DOI:** 10.3389/fnins.2015.00128

**Published:** 2015-04-22

**Authors:** Teemu Laitinen, Alejandra Sierra, Tamuna Bolkvadze, Asla Pitkänen, Olli Gröhn

**Affiliations:** ^1^Department of Neurobiology, A. I. Virtanen Institute for Molecular Sciences, University of Eastern FinlandKuopio, Finland; ^2^Department of Neurology, Kuopio University HospitalKuopio, Finland

**Keywords:** diffusion tensor imaging, traumatic brain injury, microstructure, animal models, histology

## Abstract

Traumatic brain injury (TBI) is a major cause of disability and death in people of all ages worldwide. An initial brain injury caused by external mechanical forces triggers a cascade of tissue changes that lead to a wide spectrum of symptoms and disabilities, such as cognitive deficits, mood or anxiety disorders, motor impairments, chronic pain, and epilepsy. We investigated the detectability of secondary injury at a chronic time-point using *ex vivo* diffusion tensor imaging (DTI) in a rat model of TBI, lateral fluid percussion (LFP) injury. Our analysis of *ex vivo* DTI data revealed persistent microstructural tissue changes in white matter tracts, such as the splenium of the corpus callosum, angular bundle, and internal capsule. Histologic examination revealed mainly loss of myelinated axons and/or iron accumulation. Gray matter areas in the thalamus exhibited an increase in fractional anisotropy associated with neurodegeneration, myelinated fiber loss, and/or calcifications at the chronic phase. In addition, we examined whether these changes could also be detected with *in vivo* settings at the same chronic time-point. Our results provide insight into DTI detection of microstructural changes in the chronic phase of TBI, and elucidate how these changes correlate with cellular level alterations.

## Introduction

Traumatic brain injury (TBI) is a major cause of disability and death in people of all ages worldwide (Hyder et al., 2007). TBI occurs by external mechanical forces, such as falls, vehicle accidents, violence, and sports (Maas et al., 2008). The location, type, and severity of the initial injury determine the prognosis, which varies from patient to patient. This heterogeneity leads to a wide spectrum of symptoms and disabilities, such as cognitive deficits, mood or anxiety disorders, motor impairments, chronic pain, and epilepsy. After brain injury, proper early evaluation and follow-up of the progression are crucial to improve diagnosis, management, and treatment (Maas et al., 2008).

The initial assessment of primary lesions caused by the initial injury, such as hemorrhage, tissue deformation, and edema, is currently performed using computed tomography (CT) (Marshall et al., 1991), which is the gold standard for acute examination of patients after head injury. Magnetic resonance imaging (MRI) is increasingly used in subacute and chronic phases, due to the versatile MRI contrasts available to gain better insight into the secondary damage (Gallagher et al., 2007; Aquino et al., 2015). Advanced MRI techniques, such as diffusion tensor imaging (DTI), were developed for more accurate evaluation of secondary damage (Basser et al., 1994; Basser and Pierpaoli, 1996). DTI measures the magnitude and directionality of water diffusion, which depends on the tissue microstructure (Mori and Zhang, 2006). Furthermore, under pathologic conditions, DTI provides complementary information regarding tissue integrity (Song et al., 2002; Budde et al., 2011).

Previous studies of TBI patients used DTI to evaluate progressive white matter injury, which is potentially linked to poor outcome or recovery in those patients (Sidaros et al., 2008; Kumar et al., 2009). A recent study using DTI and statistical image analysis, tract-based spatial statistics (TBSS), revealed that several brain regions are affected in patients with TBI (Kinnunen et al., 2011). Trauma-induced structural alterations in the brain are detected by DTI with much higher sensitivity than conventional anatomic imaging, but little is known about the histopathology underlying these alterations.

Some previous experimental TBI studies investigated alterations in DTI parameters in rodents, followed by histologic examination (Mac Donald et al., 2007a,b; Budde et al., 2011). These studies, however, focused mainly on acute and subacute time points after injury, and the consequences of slowly progressing secondary damage to tissue microstructure have not been evaluated (Mac Donald et al., 2007a,b; Budde et al., 2011; Hylin et al., 2013).

In the present study, we investigated the detectability of secondary injury at a chronic time-point in a rat model of TBI, lateral fluid percussion (LFP) injury (McIntosh et al., 1989), using *ex vivo* DTI. Extensive histologic examination was performed to associate the imaging findings with tissue alterations at the cellular level. Additionally, we performed an *in vivo* DTI experiment at the same chronic time-point to test whether the changes could potentially serve as non-invasive markers of cellular level alterations in chronic TBI.

## Materials and methods

### Induction of traumatic brain injury by lateral fluid percussion

TBI was induced in adult male Sprague-Dawley rats (*n* = 13, 10 weeks old, weight 300–350 g, Harlan Netherlands B.V., Horst, Netherlands) by LFP injury, as described previously (Kharatishvili et al., 2006). Briefly, rats were anesthetized with a single intraperitoneal injection (6 ml/kg) of a mixture containing sodium pentobarbital (58 mg/kg), chloral hydrate (60 mg/kg), magnesium sulfate (127.2 mg/kg), propylene glycol (42.8%), and absolute ethanol (11.6%). A craniotomy (∅ 5 mm) between bregma and lambda on the left skull convexity was then performed (anterior edge 2.0 mm posterior to the bregma; lateral edge adjacent to the left lateral ridge). LFP injury was induced by a transient (21–23 ms) fluid pulse impact against the exposed intact dura using a fluid-percussion device. The impact pressure was adjusted to induce a severe injury (3.26 ± 0.07 atm). Sham-operated control animals (*n* = 7) underwent the same surgical procedures without the impact.

Following the procedures, the animals were housed in individual cages and maintained under a 12-h light/12-h dark cycle (lights on at 07:00 a.m., temperature 22 ± 1°C, air humidity 50–60%) with free access to food and water. All animal procedures were approved by the Animal Care and Use Committee of the Provincial Government of Southern Finland and conducted in accordance with the guidelines set by the European Community Council Directives 86/609/EEC.

### Tissue preparation

Six months after TBI, 7 control rats and 8 rats with TBI were deeply anesthetized and transcardially perfused according to the following protocols. Approximately half of the animals (4 controls, 4 TBI) were perfused according to the Timm fixation protocol (Sloviter, 1982). Briefly, the animals were perfused with 0.37% sulfide solution (30 ml/min) for 10 min, followed by 4% paraformaldehyde in 0.1 M phosphate buffer, pH 7.4 (30 ml/min), 4°C for 10 min. The brains were removed from the skull, post-fixed in 4% paraformaldehyde in 0.1 M phosphate buffer for 6 h, and then washed in 0.9% NaCl for at least 24 h before MRI. The remaining animals (3 controls, 4 TBI) were transcardially perfused using a pH-shift fixation protocol (Savander et al., 1996). The rats were perfused with 0.9% NaCl (30 ml/min) for 2 min, followed by 4% paraformaldehyde in 0.1 M sodium acetate buffer [NaC_2_H_3_O_2_, pH 6.5 (30 ml/min), 4°C] for 10 min, and then with 4% paraformaldehyde in 0.1 M sodium borate buffer [Na_2_B_4_O_7_·10H_2_O, pH 9.5 (30 ml/min), 4°C] for 15 min. Before *ex vivo* DTI, the brains were removed from the skull, post-fixed in 0.1 M sodium borate for 6 h, and then washed in 0.9% NaCl for at least 24 h before MRI. During the measurements, they were immersed in perfluoro polyether (Fomblin® LC08, Solvay Solexis, Milan, Italy) to avoid signal from the solution.

### *Ex vivo* DTI

For *ex vivo* DTI, brains from control rats and rats with TBI were scanned in a vertical 9.4 T/89 mm magnet (Oxford Instruments PLC, Abingdon, UK) interfaced to a DirectDrive console (Varian Inc., Palo Alto, CA, USA) using a quadrature volume RF-coil (diameter 20 mm; Rapid Biomedical GmbH, Rimpar, Germany) as the transmitter and receiver. Data were acquired using a three-dimensional (3D) spin echo sequence with two refocusing pulses (*TR* = 1 s, *TE* = 60 ms, data matrix 256 × 74 × 56, FOV 29.3 × 17 × 12.8 mm^3^). To compensate for long-duration constant eddy currents, monopolar diffusion gradient pairs with opposite polarity for each pair were placed around each refocusing pulse. Six 3D images with diffusion weighting (diffusion time = 17 ms, *b*-value = 1000 s/mm^2^) in six uniformly distributed directions (Basser and Pierpaoli, 1998) and one image without diffusion weighting were obtained. Two averages were acquired, leading to a total scanning time of 16 h/brain. This type of protocol produces high signal-to-noise images, making accurate estimation of DTI metrics possible from only six orthogonal diffusion directions. The temperature variation of the sample during the DTI measurement period was less than ±0.25°C in separate experiments under identical conditions.

### Histology to verify the *ex vivo* DTI findings

#### Tissue processing

After *ex vivo* imaging, brains were washed in 0.9% NaCl for at least 2 h at 4°C and then placed in a cryoprotective solution containing 20% glycerol in 0.02 M potassium phosphate-buffered saline (pH 7.4) for 36 h. The brains were then frozen in dry ice and stored at −70°C until sectioning. The brains were sectioned in the coronal plane (30 μm, 1-in-5 series) using a sliding microtome. The first series of sections was stored in 10% formalin and the remaining series was stored in a cryoprotectant tissue-collecting solution (30% ethylene glycol, 25% glycerol in 0.05 M sodium phosphate buffer) at −20°C until further processing.

#### Staining for assessment of neurodegeneration, gliosis, myelination, iron deposits, and calcifications

The first series of sections was stained with thionin (Nissl) to assess the cytoarchitectonics, gliosis, and neurodegeneration in different brain areas. The second series was stained with gold chloride solution to assess the myelinoarchitectonics, damage to myelinated axons, and also plasticity in myelinated pathways (Laitinen et al., 2010). Sections from the third series were stained with alizarin red to detect calcification, which occurs particularly in the thalamus (Mäkinen et al., 2008). Finally, selected sections were stained with Perl's method to confirm the presence of iron from post-TBI bleeds within the tissue.

The severity of neurodegeneration and loss of myelinated axons, and the presence of calcification and iron deposits were semi-quantitatively scored from 0 to 3: none (score 0), mild (score 1), moderate (score 2), and severe (score 3) in rats with TBI as compared with controls (representative examples of scoring are shown in **Figure 3**). Finally, we calculated the overall damage score for each animal as the sum of individual scores based on each type of staining.

### *In vivo* DTI

We next assessed whether the histologically verified *ex vivo* DTI changes in the gray and white matter could also be detected using *in vivo* DTI. For this, TBI was induced in another group of male Sprague-Dawley rats (*n* = 9, 10 weeks old, weight 300–350 g) with LFP injury (3.42 ± 0.10 atm). Sham-operated rats were used as controls (*n* = 3). Six months later, *in vivo* DTI was performed in a horizontal 9.4 T/31 cm magnet interfaced to a Varian DirectDrive console (Varian Inc.). An actively decoupled transmitter volume RF-coil (diameter 72 mm)—4 channel surface receiver RF-coil optimized for rat head (Rapid Biomedical GmbH) was used for the acquisitions. The animals were anesthetized with 1.4% isoflurane in N_2_O/O_2_ (70%/30%) and fixed in a stereotaxic holder. Normal body temperature was maintained with a heating pad. For data acquisition, we used a 3D-segmented spin-echo EPI with a navigator echo for phase correction, a Nyquist ghost minimization scheme similar to by van der Zwaag et al. (2009), and outer volume suppression (*TR* = 2 s, *TE* = 45 ms, 2 segments, data matrix 128 × 64 × 64, FOV 25 × 15 × 15 mm^3^, 1 average). Six images with diffusion weighting applied to six uniformly distributed directions (Δ = 16 ms, δ = 5 ms, *b*-value = 1000 s/mm^2^) and one image without diffusion weighting were acquired for *in vivo* DTI (Basser and Pierpaoli, 1998). The total acquisition time for DTI data was 2 h.

### *Ex vivo* and *in vivo* DTI data analysis

*Ex vivo* DTI data were zero padded to 256 × 148 × 112 point prior Fourier-transformation leading to an interpolated spatial resolution of 115 × 115 × 115 μm^3^. *In vivo* DTI data were first corrected for Nyquist ghosting as described by van der Zwaag et al. (2009). Next, two consecutive k-space lines in the second phase encoding direction were added together to achieve a higher signal-to-noise ratio in the images, leading to a data matrix of 128 × 64 × 32 and spatial resolution of 195 × 234 × 469 μm^3^. All data were corrected for residual eddy current distortions with affine linear alignment of the diffusion-weighted images to the image without diffusion weighting with FMRIB's Linear Image Registration Tool (FLIRT) (Jenkinson and Smith, 2001; Jenkinson et al., 2002), which is included in FMRIB Software Library software (http://www.fmrib.ox.ac.uk/fsl/). Following eddy current corrections, maps of the diffusion tensor eigenvalues (λ_1_, λ_2_, λ_3_), trace of the diffusion tensor [trace(D)], and fractional anisotropy (FA), as well as directionally encoded color maps, where the color of each voxel is defined by the orientation of its diffusion tensors primary eigenvector (V_1_) and the intensity of each voxel is proportional to FA (Pajevic and Pierpaoli, 1999), were created using DTI-Studio 2.40 (https://www.mristudio.org/). From the eigenvalue maps, maps of axial diffusivity (D_||_ = λ_1_) showing the diffusivity parallel to the direction of the principal eigenvector and radial diffusivity (D_⊥_ = (λ_2_ + λ_3_)/2) describing the diffusivity perpendicular to the principal eigenvector were created, and from the trace(D)-map a map of mean diffusivity (MD = trace(D)/3) was determined using in-house built MATLAB (2010a, Mathworks, Natick, MA, USA) code and the region of interest (ROI) analysis was performed using an in-house built MATLAB code (http://aedes.uef.fi/).

**Figure 1:**
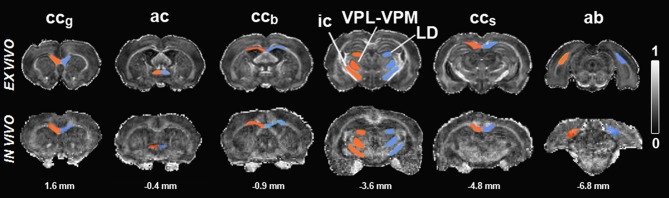
**Fractional anisotropy maps of sham-operated animals *ex vivo* (upper row) and *in vivo* (lower row)**. Regions of interest are outlined in red (ipsilateral) and blue (contralateral). Scale bar: 0 (black) to 1 (white). Abbreviations: *ac*, anterior commissure; *ab*, angular bundle; *cc_b_*, body of the corpus callosum; *cc_g_*, genu of corpus callosum; *ic*, internal capsule; *LD*, laterodorsal thalamic nuclei; *cc_s_*, splenium of corpus callosum; *VPL-VPM*, ventroposteromedial (VPM), and ventroposterolateral (VPL) nuclei. Numbers indicated below the maps correspond to the distance from bregma.

Reliable co-registration of the brain with the atlas was not possible due to large variably-sized lesions and profound atrophy in the TBI brain. Therefore, ROIs were manually outlined based on directionally encoded color maps by one of the authors (AS.) with extensive experience in neuroanatomy, both in histologic preparations and MRI. Several consecutive slices were outlined on the coronal plane to cover the entire anatomic brain volume. The selected ROIs included the angular bundle (ab), anterior commissure (ac), genu of the corpus callosum (cc_g_), body of the corpus callosum (cc_b_), splenium of the corpus callosum (cc_s_), internal capsule (ic), laterodorsal thalamic nuclei (LD), and a region comprising the ventroposteromedial (VPM), and ventroposterolateral (VPL) nuclei. The same ROIs were selected for analysis from both the *ex vivo* and *in vivo* data (Figure 1).

### Histology from *in vivo* samples

We also examined histologic sections from *in vivo* group to confirm that the neurodegeneration and loss of myelinated axons, as well as the presence of calcification and iron deposits, were comparable between the two groups.

### Statistical analysis

Data were analyzed using IBM SPSS 19.0 for Windows (SPSS Inc., Chicago, IL, USA). Data for controls and rats with TBI were compared using the Mann–Whitney *U*-test. Interhemispheric differences were compared using the Wilcoxon test. Results are expressed as mean ± standard deviation (SD). A *p*-value of less than 0.05 was considered statistically significant.

## Results

### Mortality and location and extent of cortical lesions after LFP injury

Mortality in the group of rats prepared for *ex vivo* study was 38% (5/13) and for the *in vivo* study, 44% (4/9; *p* = 0.779, χ^2^ test). As assessed from histologic preparations, the location of the injury was consistent in all of the rats after TBI in both the *ex vivo* and *in vivo* groups: cortical lesions were observed between approximately −2 and −6 mm from bregma.

### Abnormalities in white matter tracts after LFP injury

#### Corpus callosum

As the severity and extent of cortical damage at different rostrocaudal levels varied remarkably between the animals, we assessed whether the damage to the corpus callosum varied accordingly. Histologic and DTI analyses were conducted separately in the genu (cc_g_), body (cc_b_), and splenium (cc_s_) of the corpus callosum (Figure 1).

#### Genu of the corpus callosum

There were no detectable abnormalities in DTI or histology (Tables 1–**3**).

**Table 1:**
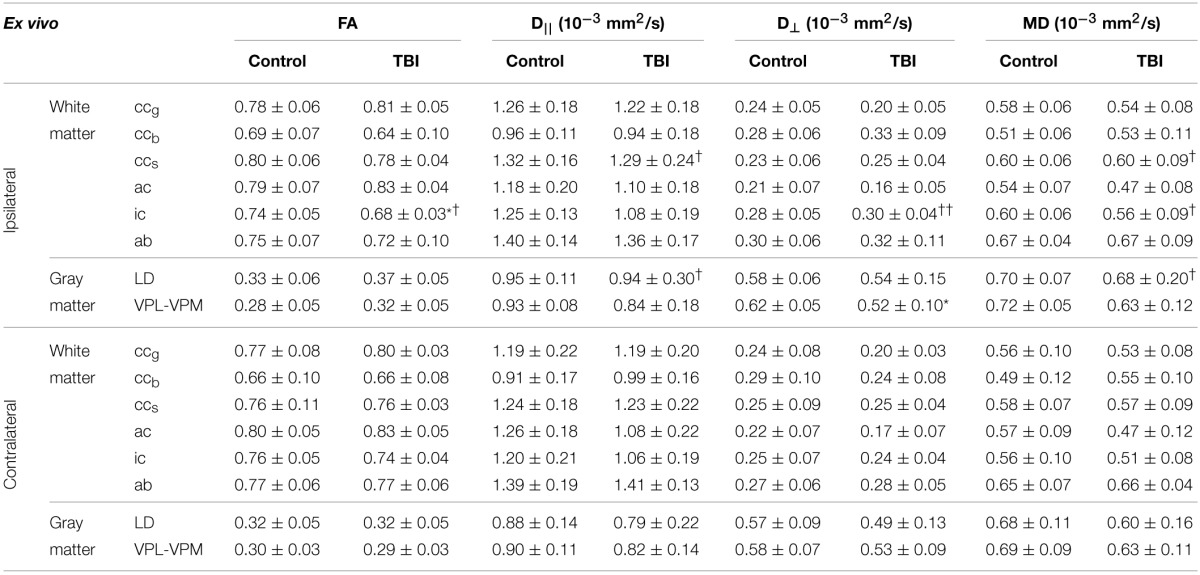
**Fractional anisotropy (FA), and axial (D_||_), radial (D_⊥_), and mean (MD) diffusivities from ipsilateral and contralateral hemispheres in controls (*n* = 7) and rats after TBI (*n* = 8) obtained using *ex vivo* DTI**.

Statistical significances are ^*^p < 0.05 comparing the same hemisphere in controls and rats after TBI (Mann–Whitney U-test) and ^†^p < 0.05 and ^††^p < 0.01 comparing ipsilateral and contralateral structures within the same animal (Wilcoxon test).

*Abbreviations: ac, anterior commissure; ab, angular bundle; cc_b_, body of the corpus callosum; cc_g_, genu of corpus callosum; ic, internal capsule; cc_s_, splenium of corpus callosum; LD, laterodorsal thalamic nuclei; VPM, ventroposteromedial nucleus; VPL, ventroposterolateral nucleus*.

#### Body of the corpus callosum

##### Ex vivo DTI

The cc_b_ showed no changes in any of the parameters analyzed, ipsilaterally or contralaterally (Table 1).

##### Histology from ex vivo DTI samples

**Figure 2:**
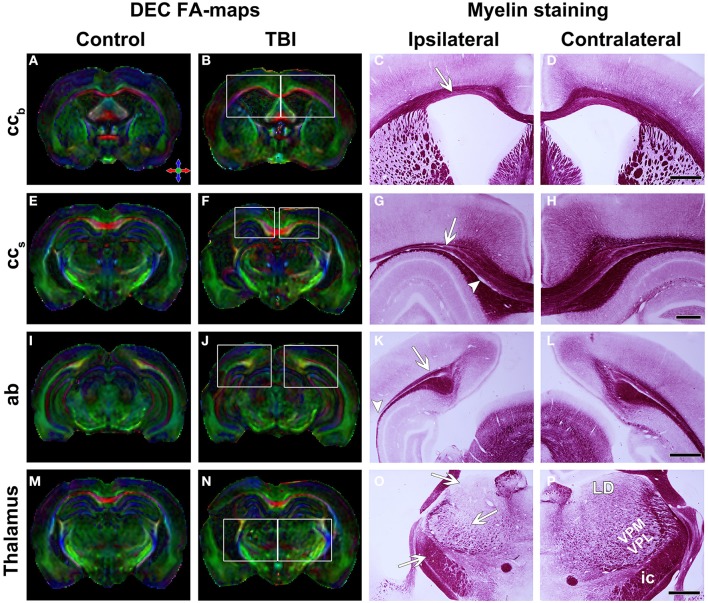
**Directionally encoded color (DEC) FA-maps of a sham-operated (A,E,I,M) and a TBI (B,F,J,N) animal**. White squares indicate regions in the photomicrographs of myelin staining. Color coding in **(A)** *red* medial-lateral, *green*, rostral-caudal and *blue* dorsal-ventral. Representative photomicrographs of myelin-stained sections of the ipsilateral **(C,G,K,O)** and contralateral **(D,H,L,P)** hemisphere of a TBI animal. Panels **(A–D)** show the body of the corpus callosum. White arrow indicates the thinning of this structure. Panels **(E–H)** show the splenium of the corpus callosum with thinning of the structure (white arrow) and loss of myelinated axons (white arrowhead). Panels **(I–L)** show the angular bundle. White arrow indicates the loss of myelinated axons and white arrowhead the disruption of this bundle. Panels **(M–P)** show the thalamus. In **(G)**, the generalized cell loss in the thalamus is observed. White arrows indicate the loss of white matter in the laterodorsal thalamic nucleus, ventroposteromedial and ventroposterolateral nuclei and internal capsule. Abbreviations: *ab*, angular bundle; *cc_b_*, corpus callosum; *ic*, internal capsule; *LD*, laterodorsal thalamic nucleus; *cc_s_*, splenium of the corpus callosum; *VPL-VPM*, ventroposteromedial (VPM), and ventroposterolateral (VPL) nuclei. Scale bars: 1 mm **(D,L,P)** and 500 μm **(H)**.

**Figure 3:**
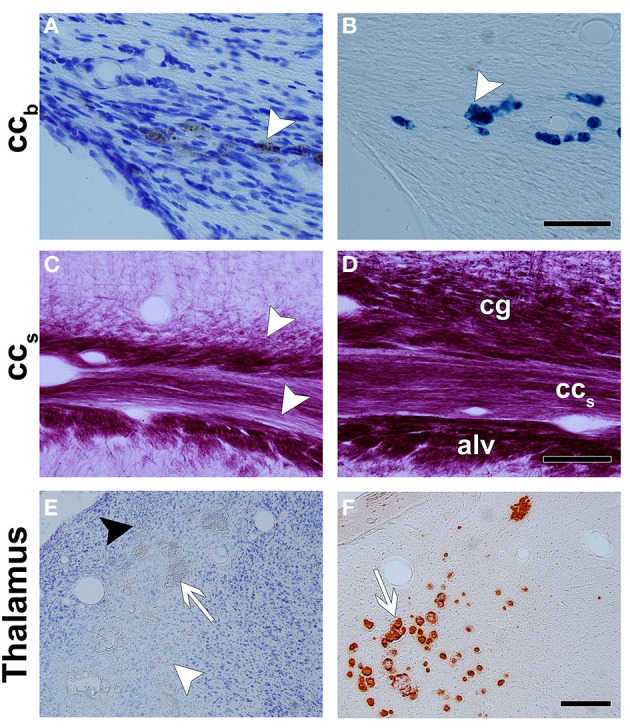
**Representative photomicrographs of Nissl- (A,E), Perl's- (B), myelin- (C,D), and alizarin- (F) stained sections**. The corpus callosum shows bleeding with a high iron content between the axons, which are dark in Nissl staining (white arrowhead in **A**) and intense blue in Perl's staining (white arrowhead in **B**). **(C,D)** are photomicrographs of a myelin-stained section of the ipsilateral and contralateral hemispheres in a TBI animal, respectively. There is a loss of myelinated axons in the splenium of the corpus callosum and in adjacent areas, the cingulum and the alveus. In the thalamus, neurodegeneration (white arrowhead), gliosis (black arrowhead), and calcification (white arrow) are observed in Nissl-stained sections **(E)**. Alizarin-stained sections show calcifications in intense red (white arrow in **F**). Abbreviations: *alv*, alveus; *cc_b_*, body of the corpus callosum; *cg*, cingulum; *cc_s_*, splenium of the corpus callosum. Scale bars: 50 μm **(A,B)**, 100 μm **(C,D)**, and 300 μm **(E,F)**.

In sections that were sampled from the level of the cc_b_ analyzed in *ex vivo* DTI, we observed remarkable thinning of the structure, reflecting the loss of myelinated axons (Figures 2A–D). Myelin loss was extensive ipsilaterally in all the rats (*n* = 8), and loss was moderate to severe (score 2 or 3) in 6 of 8 animals (Table 2). Myelin loss was also observed contralaterally in 7 of 8 rats, and loss was moderate to severe in 2 of 8 animals (Table 2). Iron accumulation was observed ipsilaterally in 3 of 8 rats with TBI (Table 2; Figures 3A,B). Although overall thinning of the cc_b_ was observed, the actual structure on the ipsilateral side remained similar to that on the contralateral side; myelinated axons run parallel along the tracts, which might explain the lack of significant findings in *ex vivo* DTI data.

**Table 2:**
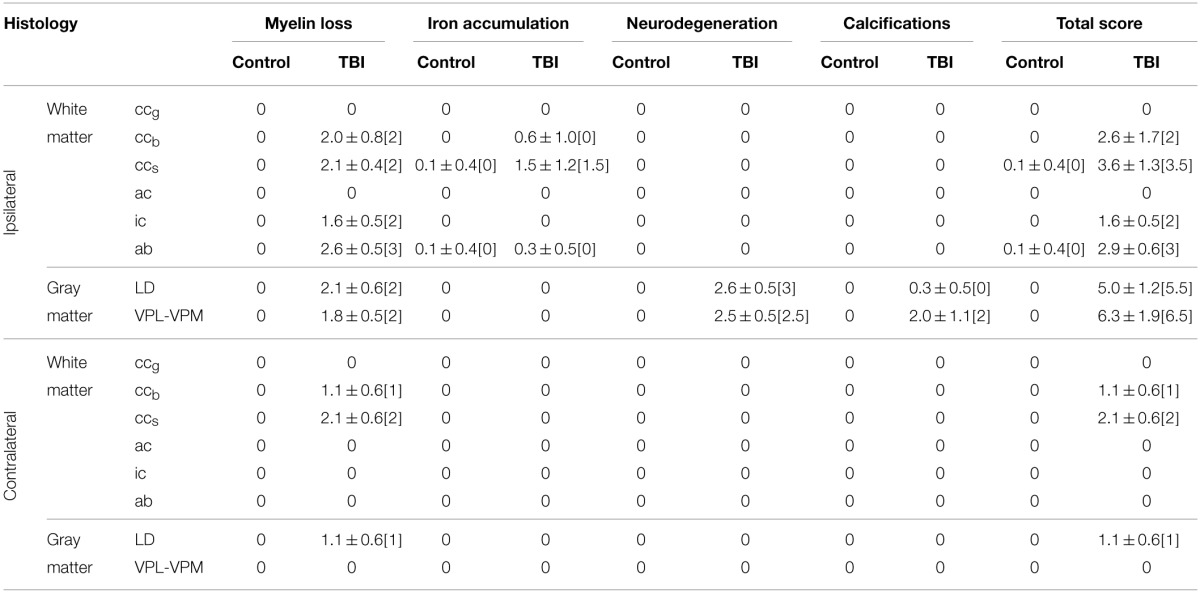
**Semi-quantitative scores comparing controls and rats after TBI**.

*Scores indicate: no (score 0), mild (score 1), moderate (score 2), and severe (score 3) alterations in tissue. The total score is the sum of individual scores from each type of stain. Values represent the mean value ± SD [median]*.

*Abbreviations: ab, angular bundle; cc, corpus callosum; ic, internal capsule; scc, splenium of corpus callosum; LD, laterodorsal thalamic nuclei; VB, ventrobasal complex*.

##### In vivo DTI

Consistent with *ex vivo* data, none of the parameters analyzed showed any statistically significant changes (Table 3).

**Table 3:**
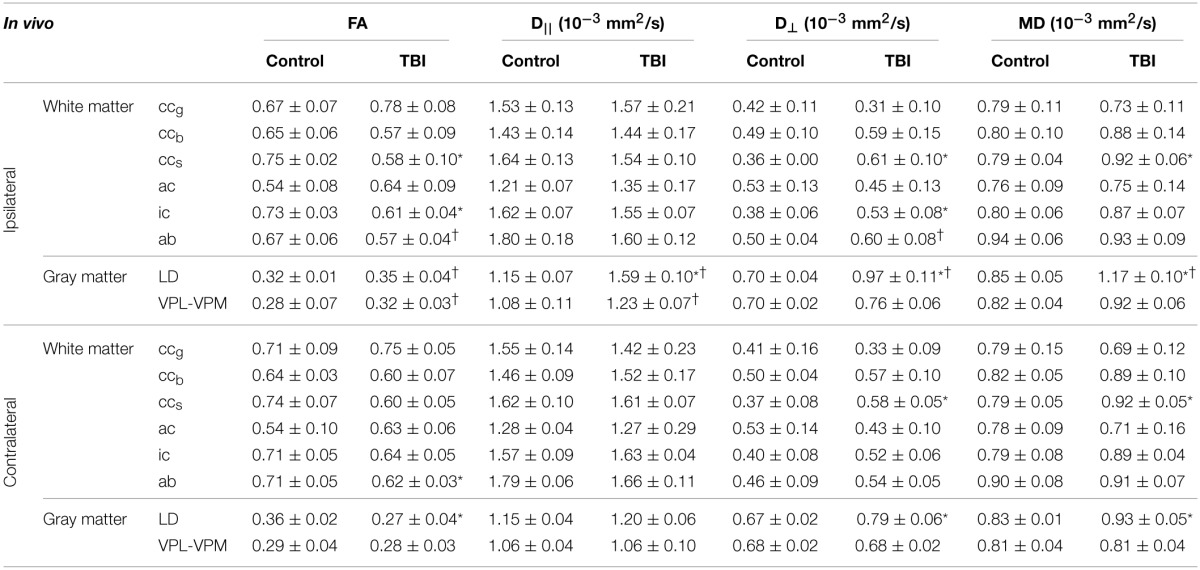
**Fractional anisotropy (FA), and axial (D_||_), radial (D_⊥_), and mean (MD) diffusivities from ipsilateral and contralateral hemispheres in controls (*n* = 7) and rats after TBI (*n* = 8) obtained using *in vivo* DTI**.

Statistical significances are ^*^p < 0.05 comparing the same hemisphere in controls and rats after TBI (Mann–Whitney U-test) and ^†^p < 0.05 comparing ipsilateral and contralateral structures within the same animal (Wilcoxon test).

*Abbreviations: ac, anterior commissure; ab, angular bundle; cc_b_, body of the corpus callosum; cc_g_, genu of corpus callosum; ic, internal capsule; cc_s_, splenium of corpus callosum; LD, laterodorsal thalamic nuclei; VPM, ventroposteromedial nucleus; VPL, ventroposterolateral nucleus*.

##### Histology from in vivo DTI samples

Histologic analysis revealed similar myelin loss and iron accumulation ipsilaterally as observed in *ex vivo* DTI samples from injured animals.

#### Splenium of the corpus callosum

##### Ex vivo DTI

The cc_s_ did not show changes in FA or D_⊥_, but D_||_ was higher ipsilaterally than contralaterally (*p* < 0.01) in rats after TBI (Table 1; Figures 2E,F). MD increased ipsilaterally compared with the contralateral side (*p* < 0.05; Table 1).

##### Histology from ex vivo DTI samples

In histologic preparations, myelin loss was observed ipsilaterally and was moderate to severe (score 2 or 3) in all the animals (Table 2; Figures 2G,H, **3C,D**). Contralateral myelin loss was also observed in all the rats, and was moderate to severe in 7 of 8 animals (Table 2). Varying amounts of iron accumulation were detected ipsilaterally in 6 of 8 rats with TBI and in 1 of 7 controls (Table 2).

##### In vivo DTI

DTI revealed a decrease in FA ipsilaterally in rats with TBI compared with controls (*p* < 0.05; Table 3). The decrease in FA was associated with an increase in D_⊥_(*p* < 0.05) and MD (*p* < 0.05) ipsilaterally and contralaterally in rats after TBI compared with controls (Table 3). The differences between *ex vivo* and *in vivo* DTI data in this area might be due to the highly variable amounts of iron present in the tract (see Discussion).

##### Histology from in vivo DTI samples

As in *ex vivo* DTI samples, histologic preparations from *in vivo* DTI samples showed myelin loss in all the TBI rats as well as iron accumulation along this white matter structure. Similarly to *ex vivo* data, myelin loss and iron accumulation extended into the contralateral hemisphere, affecting the tissue microstructure (Table 3).

#### Anterior commissure

There were no detectable abnormalities in DTI or histology (Tables 1–3).

#### Internal capsule

##### Ex vivo DTI

FA was lower in TBI animals than in controls (*p* < 0.05), and lower ipsilaterally than contralaterally (*p* < 0.05) in TBI rats (Table 1). This was accompanied by an increase in D_⊥_(*p* < 0.01 compared with the contralateral side; Table 1). MD was increased on the ipsilateral side compared with the contralateral side (*p* < 0.05; Table 1).

##### Histology from ex vivo DTI samples

Myelin-stained sections showed myelin loss ipsilaterally in all the rats (*n* = 8) that was moderate to severe (score 2 or 3) in 5 of 8 rats (Table 2; Figures 2O,P). No iron accumulation was detected in this area (Table 2).

##### In vivo DTI

Similarly to *ex vivo* DTI, FA decreased after TBI when compared with controls (*p* < 0.05; Table 3). This decrease was accompanied by an increase in D_⊥_ (*p* < 0.05 compared to controls; Table 3).

##### Histology from in vivo DTI samples

**Figure 4:**
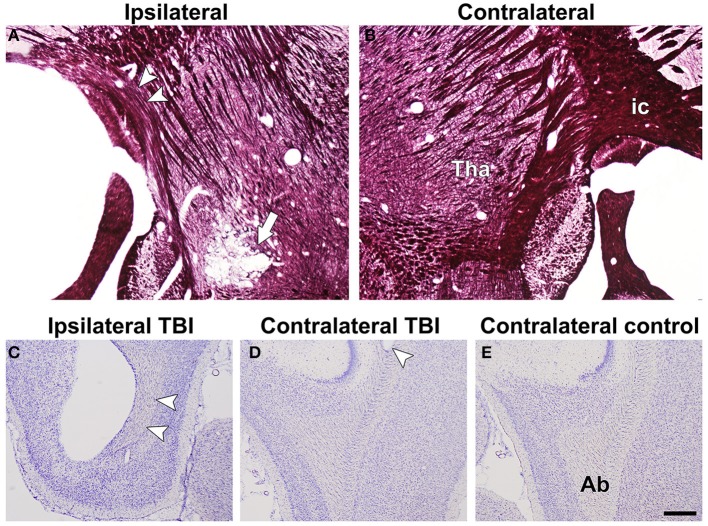
**Representative photomicrographs of myelin-stained sections of the ipsilateral (A) and contralateral (B) hemispheres of a TBI animal from the *in vivo* group**. Arrowheads point at the loss of myelinated axons in the internal capsule. The white arrow indicates calcifications in the thalamus. Representative photomicrographs of Nissl-stained sections of ipsilateral **(C)** and contralateral **(D)** hemispheres of a TBI animal, and contralateral **(E)** hemisphere of a control animal from the *in vivo* group. Arrowheads indicate damage in the angular bundle ipsilaterally and contralaterally of a TBI rat compared with controls. Abbreviations: *ab*, angular bundle; *ic*, internal capsule; *tha*, thalamus. Scale bar: 500 μm.

Histologic preparations from the *in vivo* DTI samples confirmed the changes observed in the *ex vivo* group (Figure 4A).

#### Angular bundle

##### Ex vivo DTI

None of the parameters differed from that in controls, ipsilaterally or contralaterally (Table 1; Figures 2I,J). Although FA and D_||_ appeared lower and D_⊥_ appeared elevated ipsilaterally in rats after TBI than in controls, the differences in these values did not reach statistical significance (Table 1).

##### Histology from ex vivo DTI samples

Histologic analysis revealed myelin loss ipsilaterally that was moderate to severe (score 2 or 3) in all the rats (*n* = 8; Table 2; Figures 2K,L). Iron accumulation was observed ipsilaterally in 2 of 8 rats after TBI and in 1 of 7 controls (Table 2).

##### In vivo DTI

*In vivo* DTI showed decreased FA in the ipsilateral side compared with the contralateral side (*p* < 0.05) in TBI rats (Table 3). Interestingly, FA was also decreased on the contralateral side in TBI rats compared with controls (*p* < 0.05; Table 3). D_||_ appeared lower on both the ipsilateral and contralateral sides compared with controls, although the difference was not statistically significant. D_⊥_ was increased compared to that on the contralateral side (*p* < 0.05; Table 3). The MD values remained unchanged.

##### Histology from in vivo DTI samples

Histologic preparations from *in vivo* DTI samples showed ipsilateral myelin loss as well as iron accumulation in all of the TBI rats. The cutting plane in the sections from the *in vivo* animal group allowed us to observe neighboring damage in the contralateral angular bundle (Figures 4C–E) that was difficult to visualize in the coronal sections of the *ex vivo* samples.

### Abnormalities in the gray matter after LFP injury

#### Laterodorsal thalamic nucleus

##### Ex vivo DTI

D_||_ was increased compared with the contralateral side (*p* < 0.05; Table 1), which was reflected as non-significant increasing trend in FA (Figures 2M,N). In TBI rats, MD was increased on the ipsilateral side compared with the contralateral side (*p* < 0.05; Table 1).

##### Histology from ex vivo DTI samples

In histologic sections of TBI rats, myelin loss occurred ipsilaterally in all the rats (*n* = 8), and was moderate to severe (score 2 or 3) in 7 of 8 animals (Table 2; Figures 2O,P). The contralateral side also showed myelin loss in 7 of 8 rats, which was moderate to severe in 2 of 8 animals (Table 2). We also observed extensive neurodegeneration ipsilaterally that was moderate to severe in all the rats (Table 2; Figure 3E). Calcification was observed in 2 of 8 rats after TBI (Table 2; Figure 3F).

##### In vivo DTI

FA was significantly increased (*p* < 0.05) compared with the contralateral side in TBI rats (Table 3). Additionally, the contralateral side showed a decreased FA compared with control rats (Table 3). Ipsilaterally in TBI animals, D_||_ was increased compared with both controls (*p* < 0.05) and the contralateral side (*p* < 0.05; Table 3). D_⊥_and MD were increased ipsilaterally compared with that in controls (*p* < 0.05) and contralaterally (*p* < 0.05; Table 3). These two parameters were also increased on the contralateral side compared with control rats (Table 3).

##### Histology from in vivo DTI samples

Neurodegeneration, myelin loss, and calcification were detected ipsilaterally in rats after TBI, similarly to *ex vivo* DTI samples.

#### VPM-VPL nuclei

##### Ex vivo DTI

In the VPM-VPL nuclei, there was a decrease in D_⊥_(*p* < 0.05) ipsilaterally compared with controls (Table 1). In addition, there was a non-significant trend (slightly elevated FA and MD values on the ipsilateral side of TBI rats as well as lower D_||_ values ipsilaterally and contralalerally compared with controls) that was later confirmed by *in vivo* DTI (see below and Table 1).

##### Histology from ex vivo DTI samples

Histologic sections revealed myelin loss ipsilaterally in all the rats (*n* = 8), and it was moderate in severity (score 2) in 6 of 8 animals (Table 2; Figures 2O,P). Extensive neurodegeneration that was moderate to severe (score 2 or 3) was also observed ipsilaterally in all the rats (Table 2; Figure 3E). Calcification was detected in all of the rats after TBI, which was severe (score 3) in 4 of 8 rats (Table 2; Figure 3F).

##### In vivo DTI

In *in vivo* experiments, FA was increased ipsilaterally (*p* < 0.05) in association with an increase in D_||_, compared with the contralateral side (*p* < 0.05; Table 3).

##### Histology from in vivo DTI samples

Myelin loss was observed ipsilaterally in all the TBI rats compared with contralateral side, as in *ex vivo* DTI samples (Figures 4A,B). Calcifications were also detected in most of the animals (Figures 4A,B). No iron accumulation was detected.

## Discussion

The aim of the present study was to investigate the detection of secondary injury at a chronic time-point in a rat model of TBI using *ex vivo* and *in vivo* DTI, and to determine the underlying cellular changes based on histology. To our knowledge, this is the first study using DTI to investigate chronic alterations after LFP injury. Our analysis of *ex vivo* DTI data revealed persistent microstructural tissue changes in white matter tracts, such as the splenium of the corpus callosum, angular bundle, and internal capsule. Histologic examination revealed mainly a loss of myelinated axons and/or iron accumulation in the white matter tracts. Further, gray matter areas in the thalamus exhibited increased FA after TBI related to neurodegeneration, loss of myelinated fibers, and/or calcification in the chronic phase. Findings from *in vivo* DTI as well as examination of histologic preparations from *in vivo* DTI brains corroborated the findings and indicate the potential for using these changes as non-invasive markers of specific pathologic changes.

### DTI detects persistent microstructural alterations in the chronic phase after LFP injury

Our results indicated that decreases in FA in white matter areas which is mainly associated with the loss of myelinated axons. This is consistent with findings from previous studies showing that FA is decreased in the white matter in a controlled cortical impact animal model of TBI (Mac Donald et al., 2007a,b), and in TBI patients (Sidaros et al., 2008). Massive axonal injury occurs in the corpus callosum in the acute phase after brain injury, slowing down after 4 days and ceasing within weeks (Mac Donald et al., 2007a,b). However, the body and splenium of the corpus callosum showed loss of axons in the contralateral hemisphere detected in the histology but not by DTI. Because of the continuity of the structure between both hemispheres, we expected that axonal loss would also occur contralaterally to the injury. In the body of the corpus callosum, milder changes were observed on the contralateral side (score 0 to 2) than on the ipsilateral side (score 1 to 3; Table 2). In the splenium of the corpus callosum, the severity of the axonal loss was similar on the ipsilateral side (score 2 to 3) and contralateral side (score 1 to 3; Table 2). We observed no iron accumulation on the contralateral side, however, in either the body or splenium of the corpus callosum. Although there was a similar loss of axons on the contralateral side, very little is known about the differences in microstructural and ultrastructural changes after injury to these areas as well as the limitations of the DTI detection. Further investigation is needed to study how differences in ultrastructure between the ipsilateral and contralateral hemispheres after brain may potentially influence the DTI contrast.

In white matter areas, the most consistent finding in eigenvalue-derived parameters was increased D_⊥_. This might be due to prolonged axonal degeneration (Song et al., 2002; Budde et al., 2011) and, together with decreased FA, may serve as a marker of an overall decrease in the amount of myelinated fibers. Interestingly, the changes observed in D_||_ were more variable. For example, in the splenium of the corpus callosum, D_||_ was increased, while in the internal capsule it tended to be lower. These differences between white matter tracts may be due to the morphology of the structures. The internal capsule appears to be more complex, as axons form a fan-shaped structure in contrast to the strictly parallel axonal organization in the corpus callosum and the angular bundle. The axons running through the internal capsule project to several cortical regions and are likely to undergo different changes depending on the severity of the cortical damage in the target areas. In this case, the detected changes in DTI metrics derived from a simple single tensor model have no straightforward interpretation, as the result is an average of changes caused by the damage processes to individual axons and is influenced by the orientation of these axons. In the future, more advanced diffusion MRI approaches, including high angular resolution diffusion imaging, with additional diffusion weighting directions, *b*-values, and advanced modeling, are likely to provide more comprehensive information regarding the microstructural changes in the tissue in these types of situations.

An alteration that may affect changes in the DTI parameters are the iron deposits found along the corpus callosum and the external capsule ipsilaterally to the trauma. Those deposits may come from hemorrhages or small bleeds in the acute phase that evolve to hemosiderin in the chronic phase. Iron deposits create local microscopic field gradients that may couple with diffusion field gradients and therefore locally influence diffusion metrics. This is also a likely explanation for some apparent inconsistencies in the data, such as different responses of D_||_ and D_⊥_ after TBI in *ex vivo* and *in vivo* DTI, in areas with high iron accumulation in the splenium of the corpus callosum.

In the thalamus, FA was increased in association with an increase in D_||_ and a decrease in D_⊥_. The changes observed in DTI co-localized with neurodegeneration, loss of myelinated axons, and calcification, as verified from the histology. Previous studies investigated the implications of diffuse axonal injury and neuronal atrophy in the ventrobasal complex after brain injury (Lifshitz et al., 2007). Cortical injuries may evoke anterograde/retrograde axonal degeneration within the thalamic nuclei. Therefore, neuronal death may occur in response to large, destructive cortical lesions (Ross and Ebner, 1990). More severe injuries provoke large acute axonal injuries, followed by neuronal atrophy, and consequently, neuronal death, inflammation, and accumulation of calcium (Lehto et al., 2012). It should be noted that similar to iron deposits in the corpus callosum, calcifications in the thalamus might influence DTI parameters by creating local magnetic fields.

### Chronic tissue damage after LFP injury

LFP injury is an established animal model of TBI (McIntosh et al., 1989) that mimics many of the features of human TBI, regarding both tissue damage and outcome (Graham et al., 2000; D'Ambrosio et al., 2004; Kharatishvili et al., 2007; Flygt et al., 2013; Gurkoff et al., 2013). The primary injury provokes a cortical lesion that, over time, progresses to distal areas in the brain. Chronic tissue alterations appear in the posterior part of the brain. A spatial gradient in the white matter (Mac Donald et al., 2007a), and the effect of the severity of the injury (Rutgers et al., 2008; Hylin et al., 2013) or position of the impact (Flygt et al., 2013) were previously investigated. Most studies using LFP injury focus on the acute and/or subacute phases, from a few hours to several days/weeks after injury (Graham et al., 2000; Flygt et al., 2013). The effect LFP injury on widespread myelin loss, axonal injury, inflammation, and changes in oligodendrocyte populations, as well as hemorrhages, have been extensively investigated in the acute and/or subacute phases (Dietrich et al., 1994; Flygt et al., 2013; Okubo et al., 2013). Rodriguez-Paez et al. (2005) investigated the progression of atrophy in the white and gray matter up to 1 year after LFP injury in rats. They demonstrated a progressive decrease in the number of axons in the fimbria, external capsule, thalamus, and cerebral cortex at several time-points up to 1 year using light and electron microscopy. This result is consistent with the loss of myelinated axons detected in our histologic preparations. They also observed swollen axons and inflammation as many as 6 months after TBI, which is also consistent with the negative immunoreactivity against amyloid beta precursor protein (a marker for ongoing axonal injury) in our sections (data not shown). Based on this, we conclude that our histologic observations are consistent with those of previous studies: the initial injury provokes axonal injury, neuronal damage, and hemorrhage, which progresses to a loss of myelinated axons, neuronal death, calcification, and the accumulation of hemosiderin with high iron content within the tissue several months after TBI.

### DTI as a tool to image TBI

A key aspect of this study is the possibility of measuring microstructural changes *in vivo* with possible translation to human studies. Detection of statistically significant differences between group means in DTI parameters does not mean that these changes necessarily provide a biomarker for tissue damage or outcome measures after TBI, and this must be further explored in future studies with a larger number of animals and preferably a longitudinal study design. The finding that the *in vivo* DTI data were mostly consistent with *ex vivo* results, however, is encouraging. Previous studies revealed that FA and D_⊥_ changes in white matter are not significantly influenced by paraformaldehyde fixation, while D_||_ changes may be more difficult to detect in *ex vivo* preparations (Sun et al., 2003, 2005). Another possible confounding factor between *in vivo* and *ex vivo* results is the intravoxel incoherent motion due to the capillary blood pool. This could be a noteworthy factor, especially in the thalamus where significant vascular remodeling in chronic phase of TBI has been reported (Hayward et al., 2010).

## Conclusion

Our results indicate that DTI is able to detect microstructural changes in the chronic phase of TBI. Detection of statistically significant differences between group means in DTI parameters does not necessarily indicate that these changes provide a biomarker for the tissue damage or outcome measures after TBI. This must be further investigated in in both white matter and gray matter in future studies with a larger number of animals and preferably a longitudinal study design. Whether DTI changes can serve as biomarkers for tissue damage or recovery after injury, however, requires further investigation. Experimental studies combining DTI and histologic approaches are extremely valuable for examining the origin of DTI changes at the cellular level. Overall, our data emphasize the potential of DTI as a tool to provide non-invasive information that aids in the assessment of comorbidities and alterations of the brain network in the chronic phase after brain injury.

### Conflict of interest statement

The authors declare that the research was conducted in the absence of any commercial or financial relationships that could be construed as a potential conflict of interest.

## Abbreviations

ab: angular bundle
ac: anterior commissure
cc_g_: genu of the corpus callosum
cc_b_: body of the corpus callosum
cc_s_: splenium of the corpus callosum
D_||_: axial diffusivity
D_⊥_: radial diffusivity
FA: fractional anisotropy
ic: internal capsule
LD: laterodorsal thalamic nucleus
LFP: lateral fluid percussion
MD: mean diffusivity
MRI: magnetic resonance imaging
ROI: region of interest
TBI: traumatic brain injury
VPM: ventroposteromedial nucleus
VPL: ventroposterolateral nucleus.
